# Third-Generation Cephalosporin Resistance Among Enterobacterales in Percutaneously Drained Pelvic Abscesses: A Single-Center Retrospective Cohort Study

**DOI:** 10.3390/life16091569

**Published:** 2026-09-19

**Authors:** Volkan Tasci, Onur Taydas, Ismail Ozer, Mustafa Ozdemir, Omer Faruk Topaloglu, Mehmet Ali Durmus, Muhammet Fatih Tamer, Umit Mustak, Ali Fuat Tekin, Adem Senturk, Huseyin Hatipoglu, Osman Kose, Alp Omer Canturk, Mehmet Halil Ozturk

**Affiliations:** 1 Department of Radiology, Faculty of Medicine, Sakarya University, Adapazarı 54100, Türkiye; md.volkantasci@gmail.com (V.T.); ozer815@gmail.com (I.O.); drmstfrd@gmail.com (M.O.); ofaruktopaloglu@gmail.com (O.F.T.); m.alidurmus0@gmail.com (M.A.D.); tamerfatih1461@gmail.com (M.F.T.); mushtaqomid@gmail.com (U.M.); ozturkmh@gmail.com (M.H.O.); 2 The Russell H. Morgan Department of Radiology and Radiological Sciences, Johns Hopkins University School of Medicine, Baltimore, MD 21205, USA; aftrad333@gmail.com; 3 Department of Surgical Oncology, Faculty of Medicine, Sakarya University, Adapazarı 54100, Türkiye; dr.adem.senturk@gmail.com; 4 Department of Microbiology, Faculty of Medicine, Sakarya University, Adapazarı 54100, Türkiye; huseyin.hatipoglu@gmail.com; 5 Department of Gynecologic Oncology, Faculty of Medicine, Sakarya University, Adapazarı 54100, Türkiye; koseo@sakarya.edu.tr; 6 Department of Surgery, Faculty of Medicine, Sakarya University, Adapazarı 54100, Türkiye; dr.omercanturk@gmail.com

**Keywords:** pelvic abscess, percutaneous drainage, antimicrobial stewardship, antibiotic resistance, third-generation cephalosporin resistance, Gram-negative bacteria, postoperative infection

## Abstract

**Background**: Percutaneous drainage is the cornerstone of source control for pelvic abscess, yet the microbiology and resistance burden of the drained fluid remain underexplored. **Methods**: We retrospectively analyzed data from 149 patients who underwent image-guided percutaneous drainage of a pelvic abscess at a single center. Third-generation cephalosporin resistance (3GCR) was evaluated as the primary outcome, with documented resistance across at least three antimicrobial categories (DR ≥ 3) as a secondary descriptor. **Results**: Cultures were positive in 74/101 sampled aspirates (73%), Gram-negative-predominant (73%), with *Escherichia coli* most frequent (50% of isolates). At least one *Enterobacterales* isolate was recovered from 50 patients, of whom 32 (64%) had documented resistance to ceftriaxone or ceftazidime; among patients with *E. coli*, 27/41 (66%) were 3GCR-positive. This proportion applies to the culture-positive subset with an available antibiogram. It cannot be extrapolated to all drained pelvic abscesses, since culture acquisition was selective and strongly associated with macroscopic purulence. Median abscess volume was higher in 3GCR-positive patients (213 vs. 88 cc), but the association did not reach statistical significance (OR 1.60 per 100 cc, 95% CI 0.94–2.71; *p* = 0.081; AUC 0.70) and attenuated further when restricted to adults (OR 1.49, 95% CI 0.91–2.45; *p* = 0.114). Postoperative etiology was not associated with 3GCR (14/22, 64%, vs. 9/16, 56%; OR, 1.36; 95% CI, 0.37–5.07; *p* = 0.74). Overall clinical success was 128/148 (86%), and 47/50 (94%) in the primary analysis set. **Conclusions**: In a selected subset of culture-positive pelvic abscesses, two-thirds of *Enterobacterales* were resistant to third-generation cephalosporins. Neither abscess volume nor postoperative origin predicted resistance.

## 1. Introduction

Surgical and postoperative intra-abdominal infections remain a leading cause of morbidity, prolonged hospitalization, and mortality despite advances in perioperative care, and their management has been the subject of successive international guidelines [1,2]. Pelvic abscess, whether arising from a perforated viscus, an anastomotic or biliary leak, oncologic surgery, or a spontaneous gynecologic or appendiceal source, represents a common endpoint of these processes and a frequent reason for interventional referral [2].

Effective management rests on two pillars: adequate source control and appropriate antimicrobial therapy [1,2]. The relevance of the latter has grown as antimicrobial resistance has become one of the leading global causes of death, with resistant Gram-negative bacteria, including extended-spectrum cephalosporin-resistant Enterobacterales, designated as critical-priority pathogens [3,4]. Antimicrobial stewardship, which optimizes the choice, spectrum, and duration of therapy, is therefore central to contemporary surgical infection care [1,2].

Image-guided percutaneous catheter drainage has become the first-line approach to source control for accessible pelvic collections, achieving high technical and clinical success while avoiding the morbidity of open surgery [5,6]. However, drainage is only one component of management; the accompanying antibiotic strategy is equally consequential. Empiric regimens are frequently broad and prolonged, and whether and when they can be rationally narrowed depends on knowing the causative organisms and their susceptibilities. The drainage aspirate is a uniquely informative specimen, because unlike blood cultures, it samples the infected compartment directly [6]. However, its stewardship value is rarely quantified, and it is unclear which patients harbor resistant organisms that would render standard empiric therapy inadequate. Identifying clinical or imaging predictors of resistance would allow the interventional radiologist who both images and drains the collection to contribute to antibiotic decision-making [7,8]. Although microbiological characteristics of intra-abdominal and pelvic abscesses have been described, the relationship between imaging characteristics of drained pelvic collections and antimicrobial resistance remains insufficiently characterized.

We therefore conducted a single-center retrospective cohort study of patients undergoing percutaneous drainage of pelvic abscess. Our primary aim was to identify clinical, laboratory, and CT features associated with cephalosporin-resistant Gram-negative organisms, chosen as the principal resistance outcome because it is robustly ascertainable from routinely tested agents. Secondary aims were to characterize the microbiological and resistance profile of drainage aspirates, to compare postoperative with non-postoperative collections, and to describe drainage effectiveness and outcomes. We hypothesized that (H1) complex CT features, large volume, multiloculation, gas, and thick wall would be associated with resistance, and (H2) postoperative etiology would be associated with resistance. Given the design, all analyses are considered associative and hypothesis-generating.

## 2. Materials and Methods

### 2.1. Study Design and Population

This was a single-center retrospective cohort study conducted at Sakarya University Training and Research Hospital between January 2020 and December 2025. Consecutive patients who underwent image-guided percutaneous catheter drainage of a radiologically and clinically confirmed pelvic abscess during the study period were included. A pelvic abscess was defined as a rim-enhancing fluid collection below the pelvic brim on contrast-enhanced CT, accompanied by clinical or laboratory evidence of infection. A postoperative abscess was defined as a collection arising after an operative procedure performed during the index admission or within the preceding 30 days; all other collections were classified as non-postoperative. Both postoperative and non-postoperative abscesses were eligible, with no age restriction. Seven patients aged under 18 were included; pediatric and adult patients underwent identical CT, drainage, and microbiological protocols. Because absolute abscess volume is not comparable across body size, an adult-only sensitivity analysis is reported. Patients were excluded if the collection was located outside the pelvis; if the collection was considered non-infectious, including a seroma, hematoma, lymphocele, urinoma, or necrotic tumor without evidence of infection; if drainage was performed surgically; or if clinical, imaging, procedural, or outcome data were insufficient for analysis. For repeated drainage procedures performed for the same abscess episode, only the first procedure was analyzed. The study was conducted in accordance with the Declaration of Helsinki and was approved by the Health Sciences Scientific Research Ethics Committee of Sakarya University (protocol code E-43012747-050.04-596580-488; date of approval: 16 June 2026).

### 2.2. Imaging and Drainage Technique

Diagnosis and characterization of the pelvic abscesses were based on contrast-enhanced multidetector CT examinations (Aquilion 64, Toshiba Medical Systems Corporation, Otawara, Japan). CT imaging was performed using a standard abdominopelvic protocol with a tube voltage of 120 kVp and automated tube-current modulation according to patient size. Intravenous non-ionic iodinated contrast material (Ultravist, Bayer AG, Berlin, Germany) was administered at a dose of approximately 1.5 mL/kg, up to a maximum of 120 mL, at an injection rate of 2.5–3.5 mL/s. Portal venous-phase images were acquired approximately 70–80 s after contrast administration. Axial images were reconstructed at a slice thickness of 2–3 mm, and multiplanar coronal and sagittal reformations were generated for further evaluation.

CT images were independently reviewed by two interventional radiologists, each with at least 5 years of experience in abdominal and interventional radiology. The readers were blinded to the microbiological culture and antimicrobial-susceptibility results at the time of image assessment. Readers were not blinded to the clinical indication for drainage, so knowledge of a recent operation could not be excluded; because postoperative etiology was a candidate predictor, this is stated as a limitation. Disagreements were resolved by consensus. To quantify measurement reliability, both readers independently re-evaluated a randomly selected subsample of 33 examinations, blinded to each other’s measurements and to microbiological results. Agreement for binary CT features was assessed using Cohen’s kappa, and for continuous measurements using the intraclass correlation coefficient (ICC (2, 1), two-way random effects, absolute agreement, single measure), with bootstrap confidence intervals, supplemented by Bland–Altman limits of agreement. For this analysis, abscess volume was derived from the three orthogonal diameters using the ellipsoid formula separately for each reader, so that volume reliability reflects diameter measurement rather than a separately recorded value.

The recorded CT characteristics included three orthogonal abscess diameters, the presence of internal septations or multiloculation, intralesional gas, wall thickness, attenuation measured in Hounsfield units, anatomic location, and the skin-to-collection distance. Abscess volume was estimated from the three orthogonal diameters using the ellipsoid formula: Volume = 0.52 × anteroposterior diameter × transverse diameter × craniocaudal diameter. This method provides an approximate volume and may be less accurate for highly irregular or multiloculated collections. On audit, recorded volumes agreed with this formula to within 1 cc in fewer than half of cases, indicating that it had not been applied uniformly during data collection. All volume analyses reported here therefore use volumes derived from the recorded diameters. All measurements were made retrospectively for this study on the diagnostic contrast-enhanced CT before drainage. Wall thickness was measured perpendicular to the collection margin at its thickest enhancing point, on the axial image showing the largest cross-sectional area. Attenuation was recorded from a circular region of interest of at least 1 cm^2^ placed centrally within the fluid component, avoiding the wall, gas, and dependent debris. Measurements were not repeated or averaged.

Percutaneous catheter drainage was performed under CT or ultrasound guidance using either the Seldinger or trocar technique, depending on the location, depth, accessibility, and morphology of the collection. Percutaneous anterior, lateral, or transgluteal access routes were selected based on the safest available trajectory, avoiding the bowel, urinary bladder, major blood vessels, pelvic organs, and neural structures. Drainage catheters (Skater, Argon Medical Devices, Plano, TX, USA) measuring 8, 10, or 12 French were used based on abscess size, aspirated material viscosity, and degree of internal complexity. All drainage procedures were performed by interventional radiologists with at least 5 years of procedural experience. The technique was therefore standardized in operator experience and equipment but not uniform in approach, since guidance modality, insertion technique, access route, and catheter size varied by collection. Technical variation was not analyzed as a covariate.

### 2.3. Microbiology and Resistance Definitions

The institution had no written protocol mandating a culture of percutaneous drainage aspirate during the study period. Aspirates were processed in the hospital microbiology laboratory using routine aerobic culture; anaerobic culture was requested at the discretion of the treating team rather than by protocol. After incubation, colonies were identified by mass spectrometry (VITEK MS PRIME, bioMérieux, Marcy-l’Étoile, France). Antimicrobial susceptibility testing was performed on an automated system (VITEK 2, bioMérieux) using AST-N420 cards, which replaced the AST-N325 and AST-N326 cards in 2022. Results were interpreted according to the EUCAST breakpoints current at the time of testing and were not reinterpreted retrospectively. The decision to submit aspirated material for culture was made at the discretion of the operator or referring team during routine clinical care. It was influenced primarily by the macroscopic characteristics of the aspirate. Aspirate appearance was recorded in 122 of 149 patients; among these, purulent material was documented in 73 of 88 cultured cases (83%) and in 17 of 34 non-cultured cases (50%; *p* = 0.0004), indicating that macroscopically purulent aspirate was not consistently sampled. Aspirated material was collected under sterile conditions and transported promptly to the microbiology laboratory in sterile containers. Samples intended for anaerobic culture were transferred using an anaerobic transport system and processed as soon as possible after collection. Direct Gram staining was performed, followed by inoculation onto routine aerobic and anaerobic culture media. Aerobic cultures were incubated at 35–37 °C under appropriate atmospheric conditions, while anaerobic cultures were incubated at 35–37 °C in an oxygen-free environment for at least 48–72 h, with prolonged incubation when clinically indicated.

Antimicrobial susceptibility panels were not uniform across isolates. Agents reported in the source records included up to 18 antimicrobials across different classes, as detailed in Supplementary Table S1. However, because retrospective records selectively logged documented resistant results more reliably than tested-and-susceptible results, complete susceptibility panels could not be fully reconstructed. Consequently, formal Magiorakos multidrug resistance (MDR) classification [9] was not possible. We restricted our primary analysis to documented third-generation cephalosporin resistance (3GCR). Ceftriaxone and ceftazidime were included in the Gram-negative panel throughout the study period, making 3GCR the most robustly ascertainable outcome in this dataset. However, the AST card was changed in 2022, and universal testing of both agents cannot be verified retrospectively. The Magiorakos definition of multidrug resistance (MDR) requires non-susceptibility in ≥3 tested antimicrobial categories, which cannot be validly determined when “not tested” is indistinguishable from “tested and susceptible.”

We therefore adopted third-generation cephalosporin resistance (3GCR) as the primary resistance outcome, restricted to *Enterobacterales*. Isolates with a resistant or intermediate result for ceftriaxone or ceftazidime were classified as non-susceptible. Isolates with a recorded result for only one of the two agents were classified using that agent, and the number so classified is reported. Isolates with no recorded result for either agent were treated as not evaluable and excluded rather than assumed susceptible. Non-fermenting Gram-negative organisms were excluded from the primary outcome because *P. aeruginosa* and *A. baumannii* are intrinsically resistant to ceftriaxone, and Gram-positive organisms and yeasts were excluded because they are not tested on the Gram-negative panel.

As a secondary descriptor, we recorded documented resistance across at least three antimicrobial categories (DR ≥ 3). This classification is asymmetric: an isolate that meets it fulfills the Magiorakos criterion, but an isolate that does not may simply have been tested less extensively. Because the number of agents tested per isolate was not recorded, DR ≥ 3 is reported as an exploratory descriptor rather than an MDR prevalence estimate.

### 2.4. Outcomes

The primary outcome was third-generation cephalosporin resistance (3GCR) or intermediate susceptibility, defined as documented resistance to ceftriaxone or ceftazidime. The principal clinical outcome was clinical success, defined as clinical and biochemical improvement with catheter removal and without the need for surgical drainage. Success was assessed at the time of catheter removal during the index admission. Follow-up beyond discharge was not systematic, so recurrence, readmission, and post-discharge mortality could not be reliably ascertained and are therefore not reported. Secondary outcomes included progression to surgery, length of hospital stay, catheter dwell time, and changes in C-reactive protein and white blood cell count from baseline to day 7.

### 2.5. Unit of Analysis

**Figure 1:**
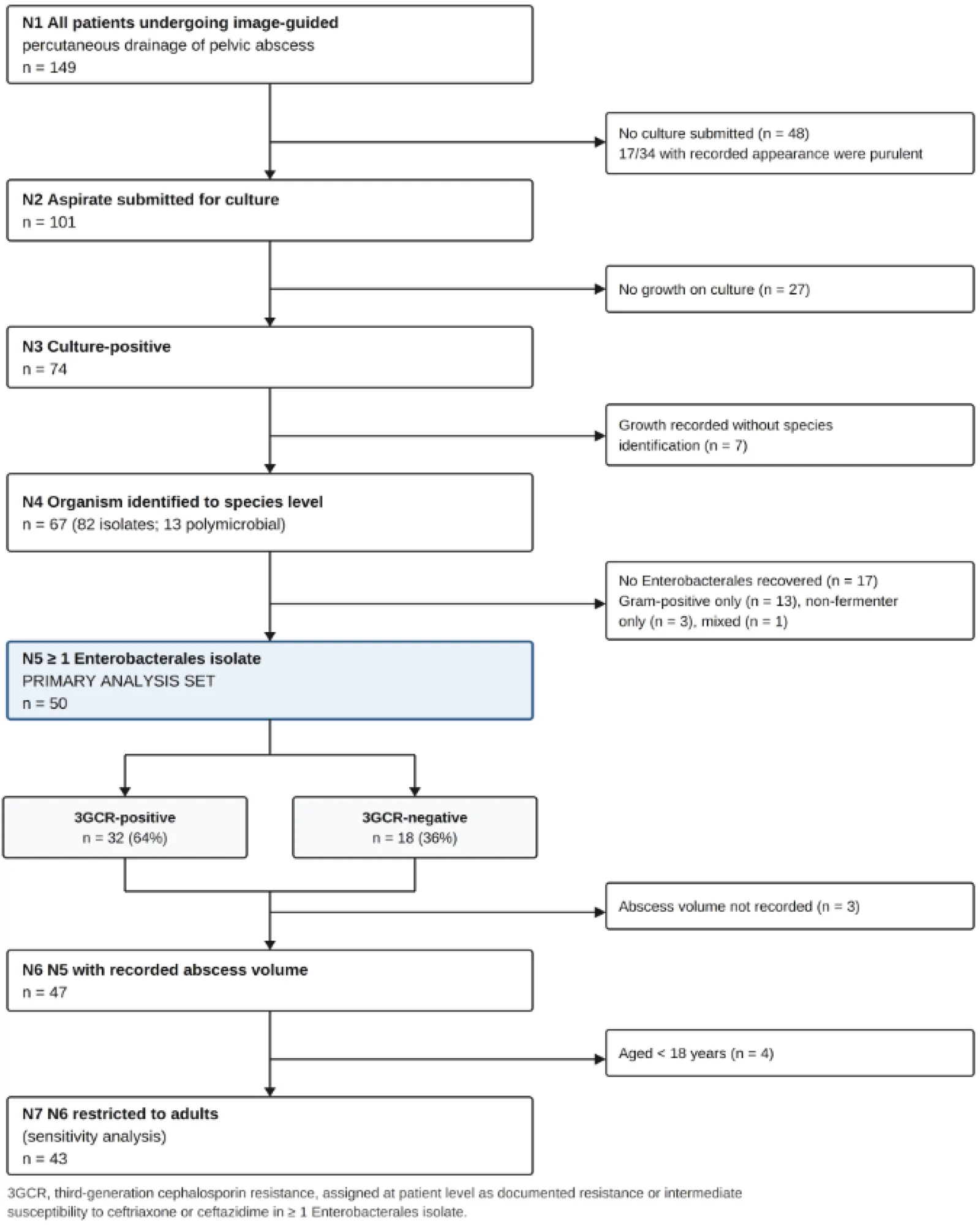
Patient flow and analytic sets. N1, all patients undergoing drainage (*n* = 149); N2, aspirate submitted for culture (*n* = 101); N3, culture-positive (*n* = 74); N4, organism identified to species level (*n* = 67); N5, at least one Enterobacterales isolate—primary analysis set (*n* = 50); N6, N5 with recorded abscess volume (*n* = 47); N7, N6 restricted to adults (*n* = 43).

The unit of analysis is the patient. Each patient contributed one drainage episode; for repeated procedures within a single abscess episode, only the index procedure was analyzed. Microbiological results were aggregated to the patient level. A patient was classified as 3GCR-positive if at least one *Enterobacterales* isolate from the index aspirate showed documented resistance or intermediate susceptibility to ceftriaxone or ceftazidime, and 3GCR-negative if at least one Enterobacterales isolate was recovered and none met that criterion. Patients from whom no Enterobacterales were recovered were not evaluable for the primary outcome and are shown as a separate branch of Figure 1. Four patients yielded two Enterobacterales isolates; the patient-level rule was applied to each. Because each patient contributes a single observation, no clustered or multilevel model was required. Where an organism-level description is reported (Table 1), the isolate is the unit, and the denominator is stated in the table header. Throughout the manuscript, patient denotes an individual in the cohort, drainage episode denotes the index percutaneous procedure, aspirate denotes the specimen obtained at that procedure, and isolate denotes a single organism recovered from an aspirate. These terms are not used interchangeably, and every table header and figure caption states which unit its denominator refers to.

### 2.6. Statistical Analysis

Continuous variables are reported as median (interquartile range) and compared using the Mann–Whitney U test; categorical variables as *n* (%) and compared using the chi-square or Fisher exact test, with denominators restricted to non-missing data. Univariable logistic regression estimated odds ratios (OR) with 95% confidence intervals for 3GCR. A parsimonious Firth-penalized model (abscess volume + postoperative etiology) was the prespecified adjusted analysis. An expanded model, additionally adjusted for malignancy and age, is reported in the sensitivity analysis as Model B in Table 5. Abscess volume and postoperative etiology were selected for analysis on the basis of the two stated hypotheses; age and active malignancy were added after univariable results were available, and we report this because post hoc covariate selection introduces optimism that penalized estimation does not correct for. Both models are underpowered for the number of parameters estimated; Firth penalization reduces small-sample bias but does not restore power, and the confidence intervals should be read as the primary description of precision. The 95% confidence interval for the AUC was estimated by 5000 bootstrap resamples. Because multiple predictors and several volume thresholds were examined, *p*-values were not adjusted for multiplicity and should be interpreted as exploratory. A two-sided *p* < 0.05 was considered nominally significant. Analyses were performed using R software, version 4.5.1 (R Foundation for Statistical Computing, Vienna, Austria). Complete-case analysis was used throughout. Item-level missingness for every variable entering the analyses is reported in Supplementary Table S1, together with the stepwise attrition from the full cohort to each analytic set and a comparison of included and excluded patients. Complete-case analysis can introduce bias when missingness is substantial or differential, and this comparison can detect such bias only for measured variables. Missingness within the primary analysis set was as follows: demographic variables, comorbidity, baseline inflammatory markers, clinical success, and resistance status were complete, while data were missing for aspirate appearance in 10%, hospital stay in 6%, albumin in 18%, etiology in 20%, and antimicrobial regimen in 34% of patients. Across the full cohort, the largest gaps were antibiotic duration (73%), antimicrobial regimen (44%), aspirate not submitted for culture (32%), etiology (27%), albumin (23%), aspirate appearance (18%), and day-7 inflammatory markers (16%).

## 3. Results

### 3.1. Cohort, Patient Flow, and Sampling

A total of 149 patients (82 female, 67 male; median age 57 years, range 7–90, including seven patients aged under 18) underwent image-guided percutaneous drainage of a pelvic abscess between 2020 January and 2025 December. An aspirate was cultured in 101 (68%), of which 74 (73%) were culture-positive; at least one Enterobacterales isolate was recovered from 50 patients, forming the primary analysis set. Etiology was recorded for 109 of 149 patients and could be classified as postoperative or non-postoperative in 103 (58 postoperative, 45 non-postoperative). Sources were appendiceal (*n* = 30, 20%), colorectal malignant (*n* = 20, 13%), gynecologic (*n* = 15, 10%), hepatobiliary or pancreatic (*n* = 9, 6%), colorectal non-malignant (*n* = 8, 5%), urologic (*n* = 7, 5%), inflammatory bowel disease (*n* = 5, 3%) and upper gastrointestinal (*n* = 3, 2%); in 12 patients (8%) the etiology field recorded a comorbidity rather than an abscess source, and in 40 (27%) no source was documented. For the postoperative subgroup, exact index procedural codes were not systematically extracted; however, the preceding surgeries corresponded to these primary pelvic pathologies (e.g., colorectal resections, appendectomies, and gynecologic interventions) (Figure 1). Baseline characteristics, overall and by third-generation cephalosporin-resistance status, are summarized in Table 2, using non-missing denominators.

Patients who had a culture sent did not differ materially from those who did not across measured demographics, comorbidity, or illness severity (all *p* > 0.15; Table 3). However, culture acquisition was selective based on fluid appearance. Aspirate appearance was recorded in 122 of 149 patients. Among these, the aspirate was macroscopically purulent in 73/88 (83%) of cultured cases compared with 17/34 (50%) of non-cultured cases (*p* = 0.0004), indicating that macroscopic characteristics strongly influenced microbiological sampling. Denominators exclude the 27 patients in whom an aspirate appearance was not recorded. In the reliability subsample (*n* = 33), agreement between readers was substantial to almost perfect for binary CT features: septation 87.1% agreement (κ = 0.74, 95% CI 0.48–0.94), loculation 87.1% (κ = 0.74, 95% CI 0.48–0.94), and intralesional gas 93.5% (κ = 0.87, 95% CI 0.66–1.00). Intraclass correlation coefficients for continuous measurements were 0.995 (95% CI 0.991–0.998) for anteroposterior diameter, 1.000 (0.999–1.000) for transverse and craniocaudal diameters, 0.999 (0.997–1.000) for derived volume, 0.990 (0.970–1.000) for wall thickness, 0.990 (0.962–1.000) for attenuation, and 0.996 (0.988–0.999) for skin-to-collection distance. These values should not be read as evidence of near-perfect measurement, because the intraclass correlation coefficient is inflated when the sample is heterogeneous: abscess diameters ranged from 20 to 165 mm and derived volumes from 17 to 1465 cc, so a genuine discrepancy of one or two millimeters contributes little to the total variance. The Bland–Altman analysis is more informative. For derived volume, the mean inter-reader difference was +1.6% with 95% limits of agreement of −6.1% to +9.4%, 97% of paired measurements fell within ±10%, and the absolute repeatability coefficient was ±26 cc. Disagreement in craniocaudal diameter was directional: in all six discordant cases, the second reader recorded the larger value (mean difference +0.45 mm), a small but systematic difference captured by the absolute-agreement form of the coefficient.

### 3.2. Microbiological and Resistance Profile

**Figure 2:**
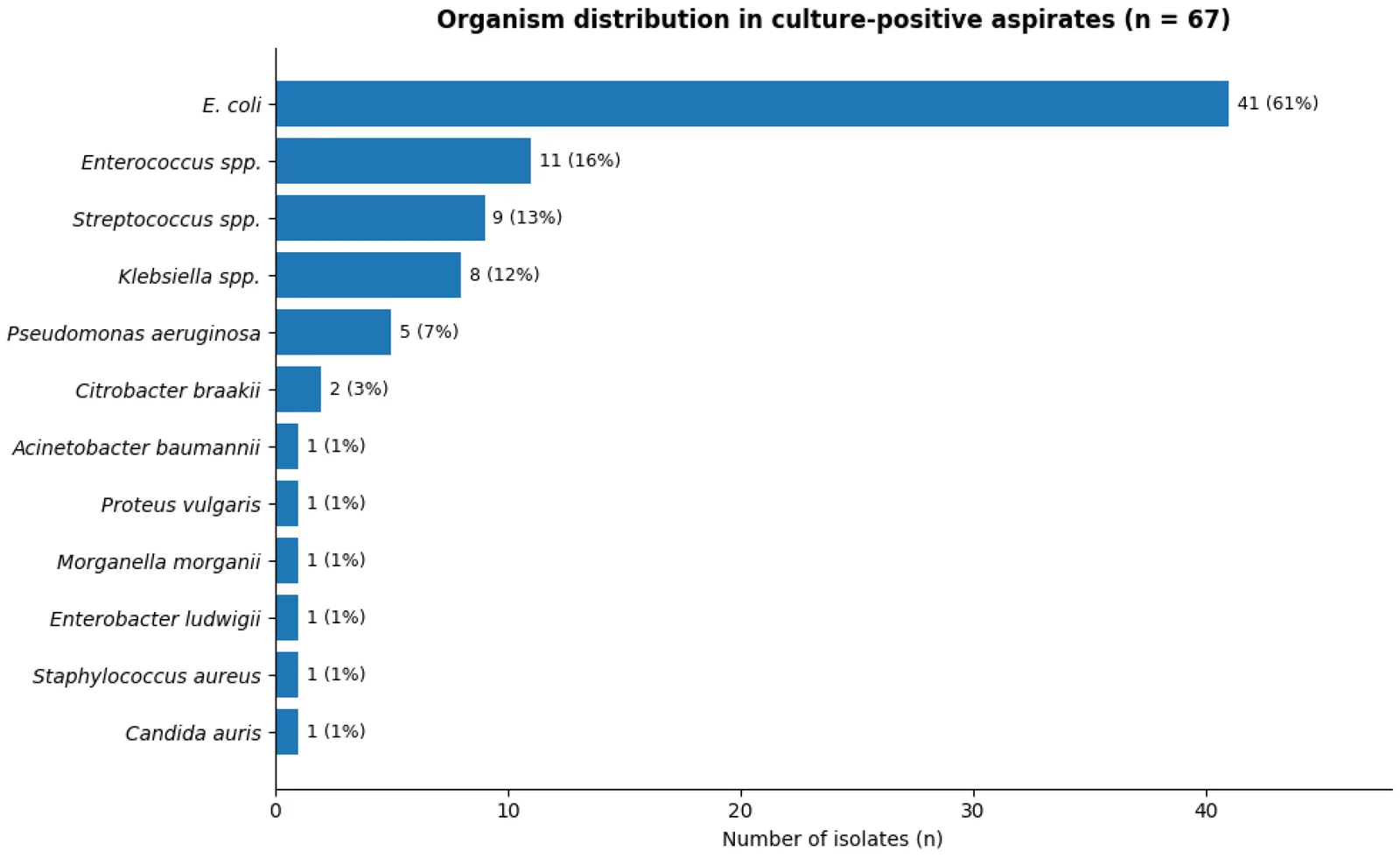
Distribution of the 82 isolates recovered from the 67 aspirates with species-level identification (N4). Percentages are of isolates and sum to 100%. Gram-negative organisms accounted for 60 isolates (73%), of which 54 were Enterobacterales, and 6 were non-fermenters. In a further 7 culture-positive aspirates, growth was recorded without species identification.

Cultures were positive in 74/101 (73%) and showed no growth in 27 (27%). An organism was identified to species level in 67 aspirates, yielding 82 isolates; 13 aspirates (13/67, 19%) were polymicrobial, 11 yielding two organisms and 2 yielding three. In the remaining 7 culture-positive aspirates, growth was recorded without species identification. Gram-negative organisms predominated (60/82 isolates, 73%), of which 54 were *Enterobacterales*, and 6 were non-fermenters. *E. coli* was the single most common isolate (41/82, 50%), followed by *Enterococcus* spp. (11, 13%), *Streptococcus* spp. (9, 11%) and *Klebsiella* spp. (8, 10%) (Figure 2). At least one *Enterobacterales* isolate was recovered from 50 patients, of whom 32 (64%) met the criterion for third-generation cephalosporin resistance; among patients with *E. coli*, 27/41 (66%) were 3GCR-positive. Documented resistance across at least three antimicrobial categories was present in 36 of 50 patients (72%), and no resistance was documented in 8 (16%), a figure that may reflect either true susceptibility or untested agents. One aspirate grew *Candida auris*, and a further aspirate grew *Acinetobacter baumannii*. Neither organism is consistent with a community-acquired enteric source. Only one of 74 positive cultures grew a *Staphylococcus* species, which argues against systematic skin contamination during percutaneous sampling.

Agent-level frequencies of documented resistance are given in Supplementary Table S1. Documented resistance was concentrated among aminopenicillins, early-generation cephalosporins, and fluoroquinolones. These frequencies are not resistance rates, because tested-and-susceptible results were not captured in the source records, and they must not be compared across agents.

### 3.3. Predictors of Third-Generation Cephalosporin Resistance

**Figure 3:**
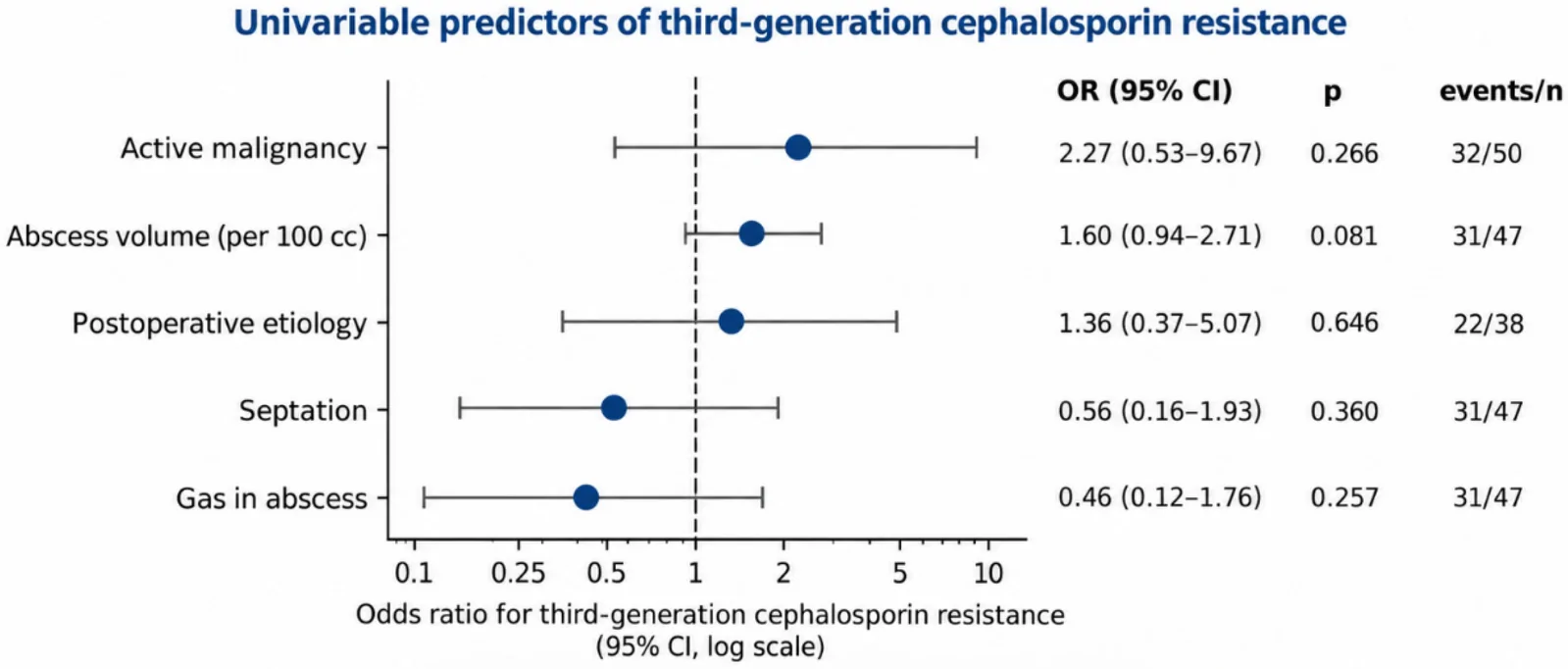
Univariable odds ratios with 95% confidence intervals for third-generation cephalosporin resistance, estimated by logistic regression. Denominators differ across predictors and are shown as events/n beside each row. The reference line is at OR = 1. No candidate predictor reached nominal significance. *p*-values are unadjusted for multiplicity and are not comparable with the group-comparison *p*-values in Table 2, which derive from the Mann–Whitney U or Fisher exact test.

In the primary analysis set (N5 = 50 patients, 32 with 3GCR), no baseline clinical variable differed significantly by resistance status (Table 2). Among the 47 patients with a recorded abscess volume (31 with 3GCR), median volume was 213 cc in 3GCR-positive and 88 cc in 3GCR-negative patients, but the univariable association did not reach statistical significance (OR 1.60 per 100 cc, 95% CI 0.94–2.71; *p* = 0.081; AUC 0.70). Restricted to adults (N7 = 43, 28 events), the estimate attenuated further (OR 1.49, 95% CI 0.91–2.45; *p* = 0.114; AUC 0.68). Median abscess volume was higher in pediatric than in adult patients (208 vs. 104 cc), so the pediatric cases clustered at the upper end of the exposure distribution; this is a further reason to interpret the volume association with caution. Gas, septation, and wall thickness were not associated with 3GCR (Figure 3). For descriptive purposes, operating characteristics at two data-derived volume cut-offs (213 cc, maximizing the Youden index, and 221 cc, giving 88% specificity) are reported in Table 4 with 95% confidence intervals. Both cut-offs were selected and evaluated in the same dataset and are therefore optimistically biased by an unquantified amount. Dichotomizing a continuous predictor also discards information, so the continuous analysis reported above is the primary one, and these values are descriptive rather than clinical decision thresholds.

In the Firth-penalized multivariable model adjusted for postoperative etiology (Table 5), neither predictor reached statistical significance (volume aOR 1.45 per 100 cc, 95% CI 0.88–2.39, *p* = 0.141; postoperative etiology aOR 1.10, 95% CI 0.28–4.30, *p* = 0.895). Estimates were essentially unchanged after additional adjustment for malignancy and age. The secondary DR ≥ 3 descriptor agreed with the 3GCR classification in 46 of 50 patients (92%).

### 3.4. Secondary Descriptor: Documented Resistance Across ≥3 Antimicrobial Categories

As a secondary descriptor, we examined documented resistance across at least three antimicrobial categories (DR ≥ 3), present in 36 of 50 patients (72%). Postoperative etiology was not significantly associated with this descriptor (17/22, 77%, vs. 9/16, 56%; OR 2.64, 95% CI 0.65–10.76; *p* = 0.289), and neither was active malignancy or polymicrobial culture (Table 6). The DR ≥ 3 descriptor agreed with the 3GCR classification in 46 of 50 patients (92%).

### 3.5. Drainage Effectiveness and Outcomes

Clinical success was achieved in 128/148 patients (86%). Twelve of 147 patients (8%) progressed to surgery, and the remaining 9 patients were managed with catheter revision or prolonged drainage. Median hospital stay was 10 days. Success did not differ between cephalosporin-resistant and susceptible cases (30/32, 94%, vs. 17/18, 94%; Fisher *p* = 1.00); however, with few failures, the study was not powered to exclude a clinically meaningful difference, so this should be read as absence of detected difference rather than proven equivalence. An antimicrobial regimen was documented for 84 of 149 patients, and a treatment duration for 40. These records were free-text, inconsistently structured, and did not reliably distinguish empirical from directed therapy or record the timing of regimen changes. They were therefore not used to assess concordance between therapy and isolate susceptibility, which remains a limitation. Therefore, clinical success reflects the combined effect of percutaneous drainage and concomitant medical therapy, and cannot be interpreted as proof of drainage efficacy independent of antimicrobial treatment. Inflammatory markers improved in both resistance strata. Among the 31 3GCR-positive patients with paired values, median C-reactive protein fell from 156 mg/L (IQR 104–200) to 30 mg/L (15–82), a median change of −107 mg/L (IQR −157 to −44; Wilcoxon *p* < 0.001); among the 18 3GCR-negative patients, it fell from 138 mg/L (96–194) to 24 mg/L (17–39), a change of −94 mg/L (−157 to −35; *p* = 0.003). The Hodges–Lehmann difference in change between groups was −11 mg/L (95% CI −71 to +50; *p* = 0.81). White cell count fell by a median of 4.3 × 10^9^/L (IQR −8.2 to −1.0) in 3GCR-positive and 3.2 × 10^9^/L (−6.7 to −1.0) in 3GCR-negative patients, with a between-group difference in change of −1.4 × 10^9^/L (95% CI −5.0 to +2.0; *p* = 0.50). Both confidence intervals are wide enough to include clinically meaningful differences in either direction, so this is an absence of detected difference rather than evidence of equivalence. Day-7 values were available for 49 of 50 patients in the primary analysis set and for 125 of 149 patients overall (84%).

## 4. Discussion

In this single-center cohort of 149 percutaneous pelvic abscess drainages, the aspirate yielded a positive culture in nearly three-quarters of cases, was Gram-negative-predominant, and carried a substantial burden of resistance, with 64% of Enterobacterales resistant to third-generation cephalosporins. Larger collections were more common among resistant cases. Still, the association did not reach statistical significance after restricting the outcome to *Enterobacterales* at the patient level, and discrimination was modest. We report this as a negative exploratory finding. While high clinical success was observed across all groups, the study was neither designed nor powered to determine whether antimicrobial resistance directly influenced clinical outcomes. We deliberately built the analysis on 3GCR rather than MDR because the available records do not permit an exact Magiorakos classification [9]; a conservative lower-bound MDR is reported as a secondary descriptor and concords with the 3GCR signal (92% agreement). The DR ≥ 3 descriptor is reported for completeness only. Differential misclassification cannot be excluded because isolates subjected to broader testing are more likely to accumulate documented resistances, so the apparent association between postoperative etiology and DR ≥ 3 may partly reflect testing intensity rather than resistance itself. We also note that ‘prior pelvic surgery’ (a remote surgical history) and ‘postoperative etiology’ (an abscess arising from recent surgery or a postoperative complication) are distinct constructs that point in opposite directions and should not be conflated. The direction of any volume–resistance relationship cannot be established from these data. Large collections may favor resistant organisms, but inadequate empirical therapy may equally allow a collection to enlarge before drainage. Under the second reading, volume is a proxy for the duration of ineffectively treated infection and possibly for prior antibiotic exposure, rather than a determinant of resistance. Neither symptom duration nor prior antimicrobial exposure was captured, so we cannot distinguish between these. A third possibility is measurement: the 95% limits of agreement for ellipsoid volume were −6.1% to +9.4%, and non-differential error of that magnitude attenuates any true association toward the null.

The *E. coli*/*Enterobacterales*-dominant spectrum is consistent with the enteric origin of most pelvic abscesses and with guideline emphasis on empiric Gram-negative and anaerobic cover pending culture [1,2]. A cephalosporin-resistance rate of this magnitude, reaching 66% among patients with *E. coli*, situates this everyday interventional-radiology population within the global escalation of resistance among Gram-negative organisms, a WHO critical priority [3,4]. If this proportion is even approximately representative locally, ceftriaxone-based empirical coverage would be inadequate in the majority of culture-positive cases at this center. We frame this as an observation about local empirical adequacy rather than as a prescribing recommendation, since neither treatment regimens nor susceptibility–treatment concordance could be assessed.

The recovery of *C. auris* from a drained pelvic collection is uncommon and, to our knowledge, has not previously been described in a percutaneous drainage series. We report it as a single observation rather than a prevalence estimate. It is relevant to the framing of this cohort: an empirical model that treats pelvic abscess as a uniformly community-acquired enteric infection would not anticipate this organism, and neither the aspirate appearance nor the CT features distinguished the case.

Our findings align with resistance-focused reports in adjacent fields. Hu et al. found a resistant organism in 50% of positive post-appendectomy drainage cultures and a culture-driven change in regimen in 42% of patients [10]; Bölük et al. reported *E. coli* dominance (52%) with ceftriaxone resistance of 56% in drained perianal abscesses, comparable to our figure of 64% among Enterobacterales [11]. In tubo-ovarian abscess, systematic per-sample culture is advocated, given an anaerobe-rich flora and rising resistance [12]. Our drainage outcomes mirror dedicated pelvic interventional-radiology series: Robert et al. reported 90% culture positivity, *E. coli* in 71% of positives, a mean diameter (68 mm) virtually identical to ours, and 97% resolution [7], and Harisinghani et al. reported 96% resolution across 154 deep pelvic abscesses [8]. Neither series reported organism-level resistance or its predictors, the gap addressed here (Table 7).

Contemporary stewardship frameworks advocate adequate initial empiric cover followed by reassessment and de-escalation once susceptibilities return [13,14,15]. Our data do not identify which regimen should be used, because antimicrobial regimens, their timing, and susceptibility–treatment concordance were not recoverable. What the data describe is the resistance burden carried by a specimen that drainage already produces. Whether drainage aspirates are routinely cultured varies between centers and is rarely audited; in this cohort, 48 of 149 drainages (32%) were performed without culture, and half of those aspirates were macroscopically purulent. We report this as an audit finding from a single center rather than as a recommendation, and we note that the value of the resistance data presented here depends entirely on the specimen having been sent [16,17,18,19].

Strengths of our study include a clinically coherent cohort drained by experienced interventional radiologists using image guidance, direct sampling of the infected compartment, a transparent patient-flow and missing-data account, a formal ascertainment-bias check, a resistance outcome chosen for valid ascertainment, and analyses matched to the data with cautious univariable inference using Firth-penalized methods and robustness analyses. Prospective, multicenter studies that re-extract complete antibiograms, capture prior antibiotic exposure, and externally validate the volume signal are the logical next step.

That 17 patients with frankly purulent aspirate had no specimen sent is a process finding rather than a study limitation, and it is the kind of gap that becomes visible only when a drainage series is assembled retrospectively. We report it because the sampling frame it defines constrains every microbiological result in this paper.

Our study had certain limitations. The microbiological dataset was generated during routine clinical practice. The overriding limitation is the inability to reconstruct complete antibiograms, as source records documented only resistant agents. Consequently, standardized MDR classification according to Magiorakos et al. [9] was impossible. We restricted our primary analysis to phenotypically documented 3GCR, and the DR ≥ 3 descriptor should be interpreted as exploratory only. Furthermore, no confirmatory phenotypic or molecular testing for ESBL, AmpC, or carbapenemase production was performed, so the mechanisms underlying the documented resistance phenotypes remain unknown. Phenotypic susceptibility testing also does not capture non-inherited mechanisms of antimicrobial survival, such as persister-cell formation, which may contribute to treatment failure independently of the reported minimum inhibitory concentrations [20]. Two changes during the study period affect interpretation. The AST card was updated in 2022, so the tested panel of agents was not identical throughout, and EUCAST breakpoints were revised for several agents during this period. Third-generation cephalosporin breakpoints for Enterobacterales were not revised, which supports 3GCR is the outcome least affected by breakpoint drift. Anaerobic cultures were not systematically characterized, and in polymicrobial aspirates (19%), no attempt was made to attribute causality to individual organisms. Thus, the presented microbiological spectrum should be viewed as a localized epidemiological snapshot rather than a generalizable resistance survey. Documented resistance to ceftriaxone and ceftazidime was concordant in 21 patients, restricted to ceftriaxone in 7, and to ceftazidime in 7, so the two agents were discordant in 14 of the 35 patients with documented resistance to either. Ceftriaxone-resistant with ceftazidime-susceptible is a recognized CTX-M phenotype; the reverse is uncommon. Symmetric discordance of this magnitude is therefore more consistent with incomplete recording than with true phenotypic divergence, and it is the clearest internal evidence that susceptible results were captured unreliably. The primary outcome was defined as resistance to either agent, which is robust to this pattern in the direction of undercounting rather than overcounting resistance. Re-extracting complete antibiograms is the highest-priority next step and would materially strengthen the resistance analysis. Unmeasured determinants of resistance. The strongest known predictors of third-generation cephalosporin resistance in Enterobacterales are prior antimicrobial exposure, recent healthcare contact, and previous colonization or infection with a resistant organism. None of these was recorded, and none could be recovered retrospectively. Two commonly cited confounders can, however, be addressed directly rather than listed as absent: no patient in the cohort was immunosuppressed (0/149), which removes immunosuppression as a confounder; and a prior drainage history was recorded for 15 patients, providing the only available proxy for prior healthcare exposure. Because postoperative patients are by definition exposed to perioperative prophylaxis and hospital contact, and because larger collections plausibly reflect longer preceding illness, both candidate predictors remain correlated with the unmeasured exposures. The associations reported here should therefore be read as markers of a patient profile rather than as independent risk factors.

Abscess culture was selective rather than routine, driven mainly by macroscopic purulence. Although the cultured and non-cultured groups were broadly similar in measured baseline clinical characteristics, this does not rule out selection bias, and the cultured subset is likely enriched for more suppurative and potentially more resistant infections. The resistance proportion reported here, therefore, applies to patients from whom an Enterobacterales isolate was recovered and cannot be extrapolated to all drained pelvic abscesses. Across the full cohort of 149 drainages, at least 32 patients (21%) had a documented third-generation cephalosporin-resistant infection; the upper bound is not identifiable, because the resistance status of the 48 patients never sampled and the 27 with no growth is unknown. No point estimate for the whole cohort is defensible.

The volume thresholds were data-derived and not externally validated; discrimination was modest (AUC 0.70, 95% CI 0.53–0.86), and the underlying continuous association did not reach statistical significance. The thresholds are reported descriptively and should not be used as a clinical rule.

Cohort heterogeneity. The cohort spans appendiceal, colorectal, gynecologic, hepatobiliary, urologic, and inflammatory bowel sources, which differ in expected flora and resistance risk. The sample is too small to model source-specific effects, and pooling them assumes a common association that we cannot test. The cohort also included seven patients aged under 18 (range: 7–90 years), for whom absolute abscess volume is confounded by body size; the adult-only analysis is therefore reported. Anaerobes were probably under-recovered (only 19% polymicrobial, no Bacteroides), tempering any empiric anaerobic recommendation [12]. Follow-up was limited to the index admission, so clinical success may be overestimated in patients who recurred after discharge. Day-7 The data were incomplete, the models were underpowered, and the *p*-values were not corrected for multiplicity.

## 5. Conclusions

Among patients with Enterobacterales recovered from percutaneously drained pelvic abscesses at our center, documented resistance to third-generation cephalosporins was present in 32 of 50 (64%). The recovered flora included healthcare-associated organisms, among them *Candida auris* and *Acinetobacter baumannii*. Larger abscess volume was more common in resistant cases, but the association did not reach statistical significance, and discrimination was modest; we report it as a negative exploratory finding. Because culture was selective, susceptibility panels were incomplete, antimicrobial exposure was unrecorded, and the analytic set was small, these results describe local epidemiology at a single center. They do not establish that abscess morphology should influence empirical antibiotic choice, and they should not be used to that end.

## Figures and Tables

**Table 1:** Microbiology and resistance summary. Isolate-level counts use the 82 isolates recovered from the 67 aspirates, with species-level identification, as the denominator; resistance is reported at the patient level in the primary analysis set (N5; *n* = 50 patients with at least one *Enterobacterales* isolate). 3GCR was defined as documented resistance or intermediate susceptibility to ceftriaxone or ceftazidime. DR ≥ 3, a secondary descriptor, was defined as documented resistance across at least three antimicrobial categories. EUCAST breakpoints were applied. ESBL and carbapenemase phenotypes were not routinely confirmed.

Parameter	Value
Aspirate sent for culture	101/149 (68%)
Culture-positive	74/101 (73%)
No growth	27/101 (27%)
Organism identified to species level	67/74 (91%)
Polymicrobial (of aspirates with species-level identification)	13/67 (19%)
Organisms (82 isolates from 67 aspirates with species-level identification)	
*Escherichia coli*	41 (50%)
*Enterococcus* spp.	11 (13%)
*Streptococcus* spp.	9 (11%)
*Klebsiella* spp.	8 (10%)
*Pseudomonas aeruginosa*	5 (6%)
*Citrobacter braakii*	2 (2%)
*Acinetobacter baumannii*	1 (1%)
*Proteus vulgaris*	1 (1%)
*Morganella morganii*	1 (1%)
*Enterobacter ludwigii*	1 (1%)
*Staphylococcus aureus*	1 (1%)
*Candida auris*	1 (1%)
Gram-negative isolates (total)	60/82 (73%)
- of which Enterobacterales	54
- of which non-fermenters	6
Resistance (patient level; N5 = 50 patients with Enterobacterales)	
3GCR—primary outcome	32/50 (64%)
Among patients with *E. coli*	27/41 (66%)
Documented resistance across ≥3 categories (DR ≥ 3)	36/50 (72%)
No resistance documented	8/50 (16%)

**Table 2:** Baseline characteristics of the cohort overall (N1, *n* = 149) and by third-generation cephalosporin resistance status within the primary analysis set (N5, *n* = 50 patients with at least one Enterobacterales isolate). Denominators reflect non-missing data and differ across columns; the overall column represents the full drained cohort, whereas the resistance columns represent only the primary analysis set. IQR, interquartile range; WBC, white blood cell count; CRP, C-reactive protein.

Characteristic	Overall (*N* = 149)	3GCR-Positive (*n* = 32)	3GCR-Negative (*n* = 18)	*p*-Value
Age, y, median (IQR)	57 (42–68)	64 (44–71)	60 (47–66)	0.42
Male sex, *n*/*N* (%)	67/149 (45)	15/32 (47)	11/18 (61)	0.39
Diabetes mellitus, *n*/*N* (%)	15/149 (10)	5/32 (16)	1/18 (8)	0.40
Active malignancy, *n*/*N* (%)	33/149 (22)	10/32 (31)	3/18 (17)	0.33
Prior drainage history, *n*/*N* (%)	15/149 (10)	3/32 (9)	1/18 (6)	1.00
Prior pelvic surgery, *n*/*N* (%)	33/149 (22)	3/32 (9)	6/18 (33)	0.055
Immunosuppression, *n*/*N* (%)	0/149 (0)	0/32 (0)	0/18 (0)	-
WBC, ×10^9^/L, median (IQR)	10.7 (8.1–14.6)	11.1 (8.8–15.5)	10.6 (7.8–14.4)	0.40
CRP, mg/L, median (IQR)	118 (61–183)	152 (103–200)	138 (96–194)	0.57
Albumin, g/L, median (IQR)	28 (25–32)	27 (24–29)	27 (23–32)	0.76
Abscess volume, cc, median (IQR)	107 (50–245)	213 (88–286)	88 (49–150)	0.03
Wall thickness, mm, median (IQR)	3.6 (2.9–4.5)	4.0 (2.9–5.0)	3.0 (2.7–3.8)	0.10
Septation present, *n*/*N* (%)	70/142 (49)	15/31 (48)	10/16 (62)	0.54
Gas present, *n*/*N* (%)	76/140 (54)	18/31 (58)	12/16 (75)	0.34
Postoperative etiology, *n*/*N* (%)	58/103 (56)	14/22 (64)	9/16 (56)	0.74
Clinical success, *n*/*N* (%)	128/148 (86)	30/32 (94)	17/18 (94)	1.00
Progression to surgery, *n*/*N* (%)	12/147 (8)	—	—	—
Hospital stay, d, median (IQR)	10 (5–19)	14 (9–27)	7 (6–16)	0.14

**Table 3:** Comparison of baseline characteristics between patients with and without a drainage-aspirate culture. Denominators are patients and reflect non-missing data; albumin was available in 80 cultured and 35 non-cultured patients, and abscess volume in 96 and 45. IQR, interquartile range; WBC, white blood cell count; CRP, C-reactive protein.

Characteristic	Cultured (*n* = 101)	Not Cultured (*n* = 48)	*p*
Age, y, median	57 (44–67)	55 (40–70)	0.78
Male sex, *n* (%)	44 (44)	23 (48)	0.73
Active malignancy, *n* (%)	22 (22)	11 (23)	1.00
Diabetes, *n* (%)	9 (9)	6 (12)	0.56
Prior pelvic surgery, *n* (%)	24 (24)	9 (19)	0.53
Prior drainage history, *n* (%)	10 (10)	5 (10)	1.00
Abscess volume, cc, median	114 (54–222)	94 (38–322)	0.81
CRP, mg/L, median	134 (69–186)	105 (56–162)	0.21
WBC, ×10^9^/L, median	11.5 (8.3–14.7)	9.8 (7.3–13.6)	0.18
Albumin, g/L, median (IQR)	28 (25–31)	27 (24–33)	0.77

**Table 4:** Operating characteristics of abscess-volume thresholds for third-generation cephalosporin. resistance (*n* = 47), reported with 95% confidence intervals. All thresholds were derived and evaluated in the same dataset and are therefore optimistically biased; the magnitude of this bias was not estimated. The values are presented in full so that the widths of the confidence intervals are apparent and are not proposed as clinical decision thresholds. CI, confidence interval; PPV, positive predictive value; NPV, negative predictive value; LR+, positive likelihood ratio.

Volume Threshold	Sensitivity (95% CI)	Specificity (95% CI)	PPV (95% CI)	NPV (95% CI)	LR+ (95% CI)
≥50 cc	0.58 (0.41–0.74)	0.75 (0.51–0.90)	0.82 (0.61–0.93)	0.48 (0.30–0.67)	2.3 (0.9–5.7)
≥200 cc	0.52 (0.35–0.68)	0.81 (0.57–0.93)	0.84 (0.62–0.94)	0.46 (0.30–0.64)	2.8 (0.9–8.1)
≥221 cc	0.45 (0.29–0.62)	0.88 (0.64–0.97)	0.88 (0.64–0.97)	0.45 (0.29–0.62)	3.6 (0.9–14.0)
≥250 cc	0.39 (0.24–0.56)	0.88 (0.64–0.97)	0.86 (0.60–0.96)	0.42 (0.27–0.59)	3.1 (0.8–12.2)

**Table 5:** Multivariable models for third-generation cephalosporin resistance by standard and Firth-penalized logistic regression, in the subset of the primary analysis set with both a recorded abscess volume and a classifiable etiology (*n* = 37). aOR, adjusted odds ratio; CI, confidence interval.

Variable	Standard aOR (95% CI)	*p*	Firth aOR (95% CI)	*p*
Model A—parsimonious (*n* = 37; 22 events)				
Abscess volume (per 100 cc)	1.62 (0.92–2.85)	0.095	1.45 (0.88–2.39)	0.141
Postoperative etiology	1.13 (0.28–4.52)	0.866	1.10 (0.28–4.30)	0.895
Model B—adjusted for malignancy and age (*n* = 37; 22 events)				
Abscess volume (per 100 cc)	1.62 (0.89–2.94)	0.116	1.43 (0.85–2.40)	0.180
Postoperative etiology	0.93 (0.21–4.12)	0.922	0.92 (0.21–3.98)	0.914
Active malignancy	2.59 (0.43–15.41)	0.296	2.17 (0.38–12.23)	0.380
Age (per 10 years)	0.94 (0.66–1.35)	0.747	0.96 (0.67–1.36)	0.804

**Table 6:** Secondary descriptor: correlates of documented resistance across at least three antimicrobial categories (DR ≥ 3) in the primary analysis set (*n* = 50, 36 with DR ≥ 3). † Postoperative denominators are the 38 patients with a classifiable etiology.

Predictor	DR ≥ 3 Documented (*n* = 36)	Not Documented (*n* = 14)	OR (95% CI)	*p*
Postoperative etiology †	17/22 (77%)	9/16 (56%)	2.64 (0.65–10.76)	0.289
Active malignancy	10/36 (28%)	3/14 (21%)	1.41 (0.33–6.10)	0.734
Polymicrobial culture	8/36 (22%)	4/14 (29%)	0.71 (0.18–2.86)	0.718

**Table 7:** Comparison of the present cohort with recent published drainage series. PCD, percutaneous catheter drainage; IAA, intra-abdominal abscess; third-generation cephalosporin resistance; n/r, not reported.

Study	Setting	*N*; Culture+	Dominant Organism/Resistance	Key Stewardship Finding
Tasci et al., 2026	Pelvic abscess, image-guided PCD	149; 73%	*E. coli* 50% of isolates; 3GCR 64% of Enterobacterales	Two-thirds of Enterobacterales 3GCR; volume not predictive
Robert et al., 2016 [7]	Deep pelvic abscess, CT transgluteal PCD	39; 90%	*E. coli* 71% of positives; resistance n/r	97% resolution; empiric therapy adapted to culture
Hu et al., 2022 [10]	Post-appendectomy IAA (pediatric), PCD	71; 48%	*E. coli*; resistant in 50% of positives	Culture changed antibiotic regimen in 42% of patients
Bölük et al., 2025 [11]	Perianal abscess (Türkiye), I&D	141; 63%	*E. coli* 52%; ceftriaxone-R 56%	Routine testing is advised to guide targeted therapy
Micheli et al., 2024 [12]	Tubo-ovarian abscess	105; n/r	Anaerobe-rich; *E. coli* ~37%	Systematic per-sample culture for adequate therapy

## Data Availability

The data presented in this study are available on request from the corresponding author. The data are not publicly available due to privacy and ethical restrictions.

## Supplementary Materials

The following supporting information can be downloaded at: https://www.mdpi.com/article/10.3390/life16091569/s1, Table S1: Frequency of documented resistance by antimicrobial agent.

## Abbreviations

The following abbreviations are used in this manuscript:

3GCR	Third-Generation Cephalosporin Resistance
DR ≥ 3	Documented Resistance across at least three antimicrobial categories
AUC	Area Under the Receiver-operating-characteristic Curve
WBC	White Blood Cell Count
CRP	C-Reactive Protein
aOR	Adjusted Odds Ratio
CI	Confidence Interval
MDR	Multidrug Resistance
ESBL	Extended-Spectrum Beta-Lactamase
IQR	Interquartile Range
ICC	Intraclass Correlation Coefficient
CT	Computed Tomography
LR+	Positive Likelihood Ratio
NPV	Negative Predictive Value
PPV	Positive Predictive Value
OR	Odds ratio
