# Supplementary material for: Third-Generation Cephalosporin Resistance Among Enterobacterales in Percutaneously Drained Pelvic Abscesses: A Single-Center Retrospective Cohort Study
Source: Life (Basel). 2026 Sep 19;16(9):1569. doi: 10.3390/life16091569 (PMC13608436; doi:10.3390/life16091569)
Supplement: Supplementary file 1 [file life-16-01569-s001.zip › life-4533565-supplementary.pdf]

Supplementary Table S1. Frequency of documented resistance among patients with a recorded antibiogram. Values are the number of patients with a resistant result for that agent, divided by the total number of patients with any recorded antibiogram. Because tested-and-susceptible results were not captured in the source records, these are not resistance rates and must not be compared across agents. A low value may reflect the absence of testing rather than susceptibility.

**Antimicrobial agent**	**All N5 patients (n = 50)**	**E. coli (n = 41)**
Ampicillin	42 (84)	34 (83)
Amoxicillin/clavulanate	20 (40)	16 (39)
Piperacillin/tazobactam	16 (32)	12 (29)
Cefuroxime	29 (58)	25 (61)
Cefoxitin	7 (14)	3 (7)
Cefixime	2 (4)	1 (2)
Ceftazidime	27 (54)	22 (54)
Ceftriaxone	26 (52)	22 (54)
Cefepime	20 (40)	17 (41)
Aztreonam	10 (20)	8 (20)
Ciprofloxacin	24 (48)	21 (51)
Levofloxacin	4 (8)	2 (5)
TMP/SMX	23 (46)	19 (46)
Gentamicin	2 (4)	0 (0)
Amikacin	1 (2)	0 (0)
Ertapenem	2 (4)	1 (2)
Imipenem	0 (0)	0 (0)
Meropenem	3 (6)	1 (2)
